# Detection of genes involved in biofilm formation in Staphylococcus aureus isolates

**DOI:** 10.3205/dgkh000267

**Published:** 2016-03-22

**Authors:** Fahimeh Nourbakhsh, Amirmorteza Ebrahimzadeh Namvar

**Affiliations:** 1 Department of Microbiology, Islamic Azad University, Shahrekord branch, Shahrekord, Iran; 2 Department of Microbiology, Faculty of Medicine, Babol University of Medical Sciences, Babol, Iran

**Keywords:** Staphylococcus aureus, MRSA, biofilm, ica gene, antibiotic resistance

## Abstract

*Staphylococcus aureus* is one of the Gram-positive pathogens causing a wide range of nosocomial infections. The present study investigates genotypic and phenotypic aspects involved in biofilm formation in methicillin-resistant *Staphylococcus aureus* strains isolated from nosocomial infections in Isfahan. A total of 110 *S. aureus* strains were collected from three major hospitals in Isfahan, the center of Iran. The antibiotic resistance pattern, phenotypes, and biofilm formation genes were studied using Congo red agar (CRA) and multiplex PCR (M-PCR). We found that 103 out of 110 samples (93.6%) were MRSA. The highest frequency of resistance was found to penicillin (89%), ciprofloxacin (87.4%), and erythromycin (86.1%). Phenotypic results showed that 53.5% were high biofilm producers, while 33.3% and 13.2% were intermediate and low biofilm producers, respectively.

*icaC* (69.3%) had the highest frequency in comparison to other intercellular adhesion (*ica*) genes, *icaD* (54.8%) was second most common.

The results show that the adherence or attachment ability and biofilm production are important for enhancing virulence factors among isolates of *S. aureus* strains.

## Zusammenfassung

*Staphylococcus aureus* ist ein Gram-positiver Erreger, der für eine Vielzahl nosokomialer Infektionen verantwortlich ist. In der vorliegenden Studie wird der Versuch eines genotypischen und phänotypischen Screenings biofilmbildender MRSA-Stämme, isoliert von Patienten mit nosokomialen Infektionen in Isfahan, unternommen. Bei 110 *S. aureus-*Stämmen, die aus den drei größten Krankenhäusern in Isfahan stammten, wurden das antibiotische Resistenzmuster sowie phänotypische und genotypische Gene für die Biofilmbildung mittels Congorot-Agar (CRA) und multiplex PCR (M-PCR) untersucht. Bei 103 von 110 Isolaten (93,6%) handelte es sich um MRSA. Die höchste Resistenzrate wurde gegen Methicillin gefunden (93,6%), gefolgt von Erythromycin (86,1%), Ciprofloxacin (87,4%) und Penicillin (89%). Phenotypisch waren 53,5% starke, 33,3% mittlere und 13,2% schwache Biofilmbilder. Im Vergleich zu anderen Intercellular adhesion (*ica*)-Genen kam *icaC* (69,3%) am häufigsten vor, gefolgt von *icaD* (54,8%).

Damit sind die Adhärenz oder das Attachment und die Biofilmbildung wichtige Virulenzfaktoren bei *S. aureus*-Stämmen.

## Introduction

*Staphylococcus aureus* is a prevalent human pathogen causing serious infections in hospitals all around the world. However, the molecular mechanisms of pathogenesis are increasing every day [1]. Under defined conditions, biofilm formation enhances the severity of *S. aureus* related infections and leads to an increased tolerance to antimicrobial agents and antibiotic resistance patterns. Biofilm formation in or on medical equipment’s and devices such as implants, increases the number and severity of nosocomial infections; thus, it is important that attempts be undertaken to remove these antibiotic resistance factors [2]. Adhesion to surfaces is the first step in producing biofilm; it is facilitated by the expression of different microbial surface components recognizing adhesive matrix molecules (MSCRAMMs), which can bind to different extracellular matrix factors such as elastin, fibronectin A and B, laminin, collagen, fibrinogen and clumping factors. These proteins can share common signal sequences for attaching to the cell wall or various surfaces [3], [4]. In a bacterial matrix, they can coat medical devices and initialize the protein production such as biofilm matrix proteins. 

This is followed by protein attachment to the bacterial surfaces, which plays an important role in *S. aureus *pathogenesis and antibiotic resistance patterns [5], [6]. In *S. aureus*, interactions with abiotic hydrophilic surfaces are controlled by polysaccharide intracellular adhesion (PIA), which is encoded by the *ica* operon (*icaABCD*). The products are also involved in the synthesis of an extracellular polysaccharide matrix which can be destroyed by available antibiofilm enzymes [7]. Polysaccharide intracellular adhesion is composed of β-1-6-linked N-acetyl glucosamine with partially deacetylated residues that surrounds human cells or medical tools and protects the microorganism against both host immune system and antibiotic treatments [8], [9], [10], [11], [12]. In this study, we evaluated the effective genes in biofilm formation of methicillin-resistant *S. aureus* isolates.

## Materials and methods

### Bacterial isolates and culture conditions

*Staphylococcus aureus* isolates were collected over a one-year period from three hospitals of Isfahan, Iran (Alzahra, Shareati and Kashani). A total of 110 *S. aureus* strains were recovered from different types of infections, including blood (n=17), decubitus ulcers (n=6), wounds (n=49), abscesses (n=3), tracheal secretions (n=25), catheters (n=4), synovial fluid (n=3) and CSF (n=3). All isolates were cultured on blood agar (Merck, Germany) and then incubated aerobically at 37°C for 48 h. After that, dubious colonies were examined using techniques for identifying *Staphylococcus* spp., such as morphology identification, catalase and coagulase production, growth on mannitol salt agar and DNase testing (Merck, Germany). Subsequently, the API-20-Staph system kit (bioMérieux, France) was used for final confirmation.

Antibiotic resistance patterns were examined by the disk diffusion method on Mueller-Hinton agar. *S. aureus* isolates were tested with methicillin (5 µg/disk), penicillin (10 ug/disk), imipenem (10 µg/disk), cefazoline (30 µg/disk), cefalotin (30 µg/disk), ceftriaxone (30 µg/disk), gentamicin (10 µg/disk), ciprofloxacin (5 µg/disk), clindamycin (2 µg/disk), azithromycin (15 µg/disk), erythromycin (15 µg/disk), mupirocin (30 µg/disk), rifampicin (5 µg/disk), tetracycline (30 µg/disk), trimethoprim (5 µg/disk), vancomycin (30 µg/disk) and nitrofurantoin (300 µg/disk) by the Kirby-Bauer disk diffusion method (MAST, Merseyside, England), according to the Clinical and Laboratory Standards Institute (CLSI) 2011. S. aureus ATCC25923 was used as the control strain. MRSA isolates were selected to undergo biofilm formation analysis [13].

### Biofilm formation (microtiter plate and CRA culture)

The biofilm production analysis was performed by cultivating the *S. aureus* strains detected in nosocomial infections on Congo Red Agar (CRA) plates, as employed and described elsewhere [14]. The CRA plates were incubated at 37°C in aerobic conditions for 24 h, and then stored at room temperature for 48 h [15]. 

The formation of reddish black colonies on CRA plates was considered as slime production. Non-slime producing strains produced smooth, pinkish-red colonies with a darkening at the center. As an alternative to the microtiter plate assay method, polystyrene plates were used, in which 20 microliters of isolates were added and incubated for 48 h at 37°C, followed by washing with phosphate-buffered saline (PBS). Finally, safranin and ethanol were used to determine biofilm-producing isolates. The absorbance was evaluated at 490 nm with an ELISA reader. These biofilm-producing isolates were selected for biofilm gene determination with molecular PCR method [16].

### DNA extraction and multiplex (M)-PCR amplification

A typical colony was cultivated in 1 ml TSB for 24 h at 37°C. The bacterial genomic DNA was extracted with a QIAGEN plasmid Minikit (Fermentas, Germany) as recommended by the manufacturer [17]. Biofilm genes determined by previously described specific primers as listed in Table 1 (Tab. 1) [11], [18]. 

## Results

We found that 103 MRSA isolates from 110 collected specimens were resistant to methicillin (MRSA). These isolates were selected for biofilm-producing evaluation with CRA medium culture and microplate titration. 

**Figure 1:**
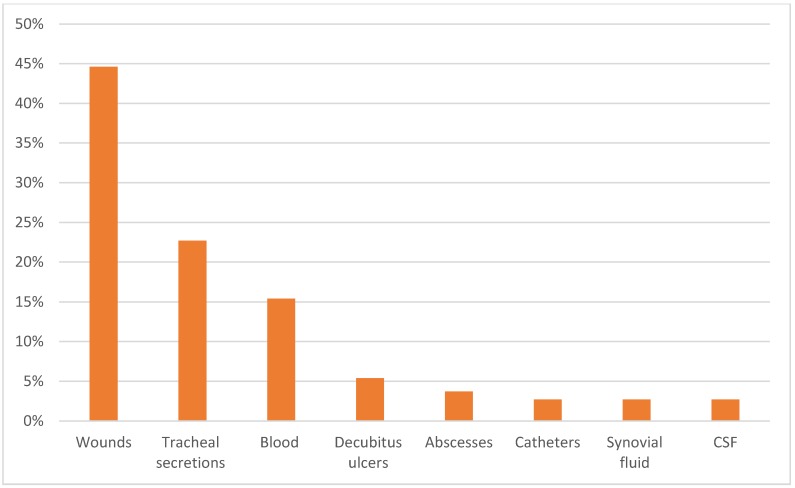
Frequency of biofilm-producing in *S. aureus* isolates by site of infection

All of the 103 isolates produced biofilm in different degrees. The most frequent resistance observed was to penicillin (89%), ciprofloxacin (87.4%), and erythromycin (86.1%). The least common resistance was belonged to nitrofurantoin (7%). The phenotypic method showed that (53.5%) of isolates were highly capable for biofilm production, while (33.3%) were intermediate biofilm producers and (13.2%) of isolates were low biofilm producers. This study demonstrated that the highest frequency of biofilm formation was found in decubitis ulcers (85%), wounds (78.2%), and tracheal secretion isolates (65.4%). The frequency of biofilm production by *S. aureus* isolates is illustrated in Figure 1 (Fig. 1).

The prevalence of the 12 genes involved in biofilm production was: *icaA* (34.2%), *icaB* (29.7%), *icaC* (69.3%), *icaD* (54.8%), *fnbA* (38.1%), *fnbB* (46.6%), *fib* (39.9%), *clfA* (41.4%), *clfB* (44.1%), *ebps* (26.5%), *cna* (18.3%), and *eno* (29.6%).

## Discussion

The mechanism of biofilm formation in *S. aureus* is not well understood, and only a limited number of studies on the expression profiles of genes involved in biofilm production exist. To understand the molecular mechanism of biofilm formation, we sought to define the detection of 12 effective selected genes in biofilm formation, including the *icaABCD* and other related genes [19], [12]. Because the *icaA* and *icaD* genes are regarded as necessary factors for intercellular adhesion, it could be assumed that these genes are only important for the formation of the bacterial multilayer in biofilm production. However, these genes are in fact associated with both slime and biofilm formation [15], [20].

Similar to our study, several other researchers have shown that formation of slime and biofilm in *S. aureus* and *S. epidermidis* is associated with the presence of *icaA* and *icaD* genes [20], [21], [22]. Production of intercellular adhesion molecules, e.g., by *icaABCD* and other genes, plays an important role in staphylococcal biofilm. Biofilm production may be the fundamental reason for the increasing antibiotic resistance of *S. aureus* strains. 

In recent investigations comparing biofilm cells with planktonic cells, the results showed that the *ica* gene can be considered necessary for the initiation of biofilm development [23]. Comparable to our observation the *icaC* gene was detected in a high number of isolates and also expressed from the *icaABCD* operon in the study by Beenken [24] and Vandecasteele [25].

However, in the present study, all of the strains harbored *ica* gene and produced slime, as detected by CRA, microplate titration, and molecular method. Slime formation as detected with CRA and microplate titration was also reported by Rohde et al. [26]. The prevalence of* clfA, clfB, cna *and* eno* genes was compared with those found in *S. aureus* strains isolated from different clinical specimens in various studies [26].

Due to our study, the percentage of *clfA* was (41.4%), as similar as Ythier study in 2012 [27]. 

According to the importance of this issue, related studies have been conducted in different regions of Iran. For example biofilm formation and antimicrobial resistance pattern in MRSA isolated from nosocomial infections and also burn patients, have been examined previously [28], [29]. A high prevalence of antibiotic resistant *S. aureus* strains was observed in this study. Thus, knowledge of the characteristics of MRSA isolates and definition of their antibiotic resistance patterns would be helpful for therapeutic decisions and useful for antibiotic therapy in infected patients. In addition to studies of *S. aureus*, the incidence of virulence genes of biofilm-producing strains of *S. epidermidis* isolates from clinical samples was studied in various provinces of Iran [30]. As opposed to previous works, the current study investigated 12 different genes from biofilm-producing MRSA strains isolated from nosocomial infections. In conclusion, the adherence or attachment ability and biofilm production of MRSA strains plays an important role in enhancing virulence factors of clinical isolates.

The prevalence of twelve genes involved in biofilm formation explains that numerous factors may be effective in different steps of biofilm production because all of the strains had capability to form biofilm in various levels but the incidence of genes was different. Further researches are considered necessary to elucidate the expression of these genes in *S. aureus* strains.

## Notes

### Competing interests

The authors declare that they have no competing interests.

## Figures and Tables

**Table 1:**
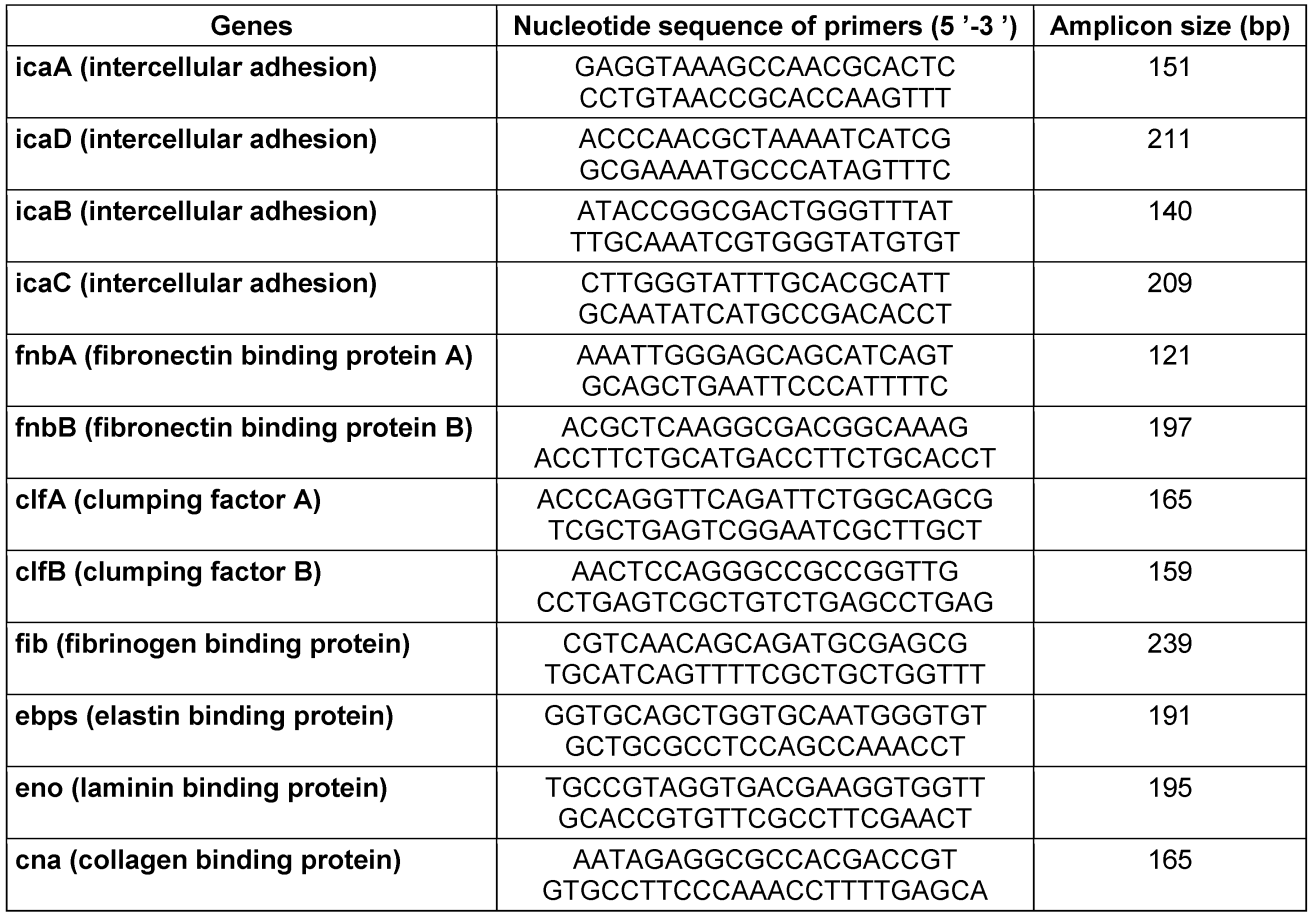
The biofilm gene primers
